# Research and application of herbal medicine in the treatment of chronic kidney disease since the 21st century: A visualized bibliometric analysis

**DOI:** 10.3389/fphar.2022.971113

**Published:** 2022-09-30

**Authors:** Yunling Xu, Jia Chen, He Wang, Ying Lu

**Affiliations:** ^1^ Zhejiang Academy of Traditional Chinese Medicine, Hangzhou, China; ^2^ Hangzhou Linping Hospital of Traditional Chinese Medicine, Hangzhou, China

**Keywords:** herbal medicine, chronic kidney disease, bibliometrics, hotspots, research status

## Abstract

**Background:** Here, a bibliometric and knowledge-map analysis was used to analyze the research status and application of herbal medicine for the treatment of chronic kidney disease (CKD). By looking for research hotspots and key topics, we provide new clues and research directions for future research.

**Methods:** Articles and reviews regarding herbal medicine in the treatment of CKD were retrieved from the Web of Science Core Collection on 23 May 2022. The R-bibliometrix, VOSviewer, and CiteSpace software were used to conduct the bibliometric and knowledge-map analysis.

**Results:** In total, 5,920 authors at 1,330 institutions from 68 countries published 1,602 papers in 355 academic journals. China is the leader and pioneer in the research and application of herbal medicine in the field of CKD treatment. Beijing University of Chinese Medicine contributed the most publications. Ping Li (China-Japan Friendship Hospital) published the most articles, while Yingyong Zhao (Northwest University) had the most cocitations. However, cooperation among countries and the research institutions is not sufficient. *Journal of Ethnopharmacology* published the most research and application of herbal medicine in the treatment of CKD and was the most commonly co-cited journal. The most influential research hotspots about herbal medicine in the treatment of CKD focused on diabetic nephropathy-related research, Balkan endemic nephropathy, and pharmacokinetic study.

**Conclusion:** Herbal medicine has a wide range of pharmacological activities and therapeutic value. The research and application of herbal medicine for the treatment of CKD, especially diabetic nephropathy, will remain a hot topic in the future.

## Introduction

Chronic kidney disease (CKD) is a multifactorial and slowly progressive condition that causes a decrease in renal function, and this condition is not effectively treated with the current therapies (Zhong et al., 2017; Kalantar-Zadeh et al., 2021). Epidemiological surveys show that the high global incidence rate of 11%–15% makes CKD a major public health problem worldwide, especially in high-income countries (Hoste et al., 2018; Lv and Zhang, 2019). The global burden of CKD is continuously increasing and is projected to become the fifth most common cause of years–of–life lost globally by 2040 (Polenakovic et al., 2021). At present, there is no specific drug or safe drug treatment for CKD in clinical, so it is urgent to discovering effective and safe new therapeutic drugs.

Numerous drugs have originated from herbs or natural substances (Geng et al., 2020; Cao et al., 2022). A large number of clinical trials and experimental studies have demonstrated that herbal medicine, either as a single natural herb or a polyherbal formulation, has an effect on protecting kidneys and improving chronic inflammation, gaining more attention for the treatment of CKD all over the world (Peng et al., 2005; Shen et al., 2018). Due to the specific theories and thousands of years clinical practice, herbal medicine has been confirmed to exhibit multi-target and multi-channel protective effects in CKD (Chen et al., 2018; Yang et al., 2019). With the expansion of clinical application and the rapid development of modern biomedical and technology, the research and application of herbal medicine in the treatment of CKD need a comprehensive summary and generalization to determine the research trend. New technologies and diagnostic criteria are also being introduced, Therefore, the trends and hotspots of herbal medicine in the treatment of CKD research have also changed in recent years, resulting in challenges to clinical work and researchers. Groups of clinicians and scholars have made great efforts and published many papers in this field. However, a summing-up commentary is lacking. A comprehensive summary and generalization to determine the research trend of this field is essential for the benefit of old and new participants in this field.

As an interdisciplinary science, bibliometrics is used for assessing and monitoring the progress of specific disciplines *via* mathematical methods and statistical analysis of published data (Demir et al., 2020; Li et al., 2020). Bibliometric analysis can be used to estimate the outputs and citations of countries, institutions, and authors and the keyword frequency of research hotspots and frontiers in particular fields (Luo et al., 2021). By using bibliometric techniques, this study sought to conduct a 22-year longitudinal view (2000–2021) of the evolution of the scientific literature on the research and application of herbal medicine in the treatment of CKD. The published literature was primarily analyzed using the following criteria: publication year, country, affiliation, journal, author, keyword, citation, and *H*-index. Finally, the bibliometric analysis results were combined with a traditional review conducted under the guidance of bibliometrics to demonstrate the evolution of the research and application of herbal medicine for CKD. This study was the first to conduct a statistical analysis on the literature on herbal medicine for CKD. It could help researchers determine future research strategies and provide them with useful references for funding decisions (Wang et al., 2021).

## Materials and methods

### Data collection

The Science Citation Index-Expanded database of the Web of Science (WoS) was used to obtain bibliographic data. Deviations caused by daily database renewal were prevented by retrieving and downloading all documents published between 2000 and 2021 from the WoS Core Collection (WoSCC) database on 23 May 2022. The search terms were: TOPICS = (chronic kidney disease OR CKD OR chronic renal disease OR chronic renal failure OR chronic renal insufficiency OR lupus nephritis OR chronic glomerulonephritis OR chronic nephropathy OR chronic nephritis OR tubulointerstitial nephritis OR diabetic kidney OR diabetic nephropathy OR nephrotic syndrome) AND TOPICS = (herbal medication OR herbal medicine OR herbal formulas OR herbal extract OR herbal supplement OR herbal products OR traditional medicine OR traditional herbal medicine OR traditional Chinese medicine OR Chinese medicine OR Chinese herbal decoction OR Chinese herbal medicine OR Chinese herbal preparation OR Chinese patent medicine OR patent herbal drug). The language was limited to English, and only research articles or reviews were retrieved. Two investigators (YLX and JC) independently conducted the primary data search and discussed any discrepancies. The final agreement reached a value of 0.90, indicating a substantial agreement (Landis and Koch, 1977). The data were saved and stored in download_txt format. Proceedings papers, editorial materials, meeting abstracts, early access, letters, book chapters, and corrections were excluded.

### Data analysis and visualization

Microsoft Office Excel 2019 was used to manage data and analyze annual publications. The total number of publications (NP) was used to measure productivity, and the total number of citations (NC) without self-citations was used to represent the impact. The H-index was used to evaluate the academic contribution and predict future scientific achievements. For visual analysis, all valid data collected from the WoSCC database were imported into VOSviewer (version 1.6.10) and CiteSpace (version 5.8.R3). The collaborative networks between countries, institutions, journals, and authors and the co-citation of keyword clusters were visually analyzed using VOSviewer. On the VOSviewer maps, different bubbles represent different elements, while the size of each bubble indicates the NP or cooccurrence frequency, where the larger the NP/frequency, the larger size of its bubbles. A line between two bubbles reflects the relationship, where the larger the scale of the cooperation, the thicker the connecting line is (van Eck and Waltman 2010). CiteSpace was used to analyze the research progress, investigate the research status, and hotspots, construct the dual-map overlay for journals and determine the field’s development trend (Chen 2004; Ma et al., 2021). The research development trajectory and current status of this field can be fully understood by examining keyword frequency, centrality intensity, and prominence (Romero and Portillo-Salido 2019). A coword network was constructed based on the keyword co-occurrence, in which each node represents a keyword. When two keywords appear in the same article, they form a co-occurrence relationship and are represented in the network as a single edge. A large mean value indicates that the node has significant representation in a particular subject field at a specific time. The degree of emergence suggests the collinear frequency of a node and the amount of increase in the number of cocitations over time. The greater the degree of emergence is, the more evidence that the node was a research hotspot during a given period.

## Results

### Publication overview

**FIGURE 1:**
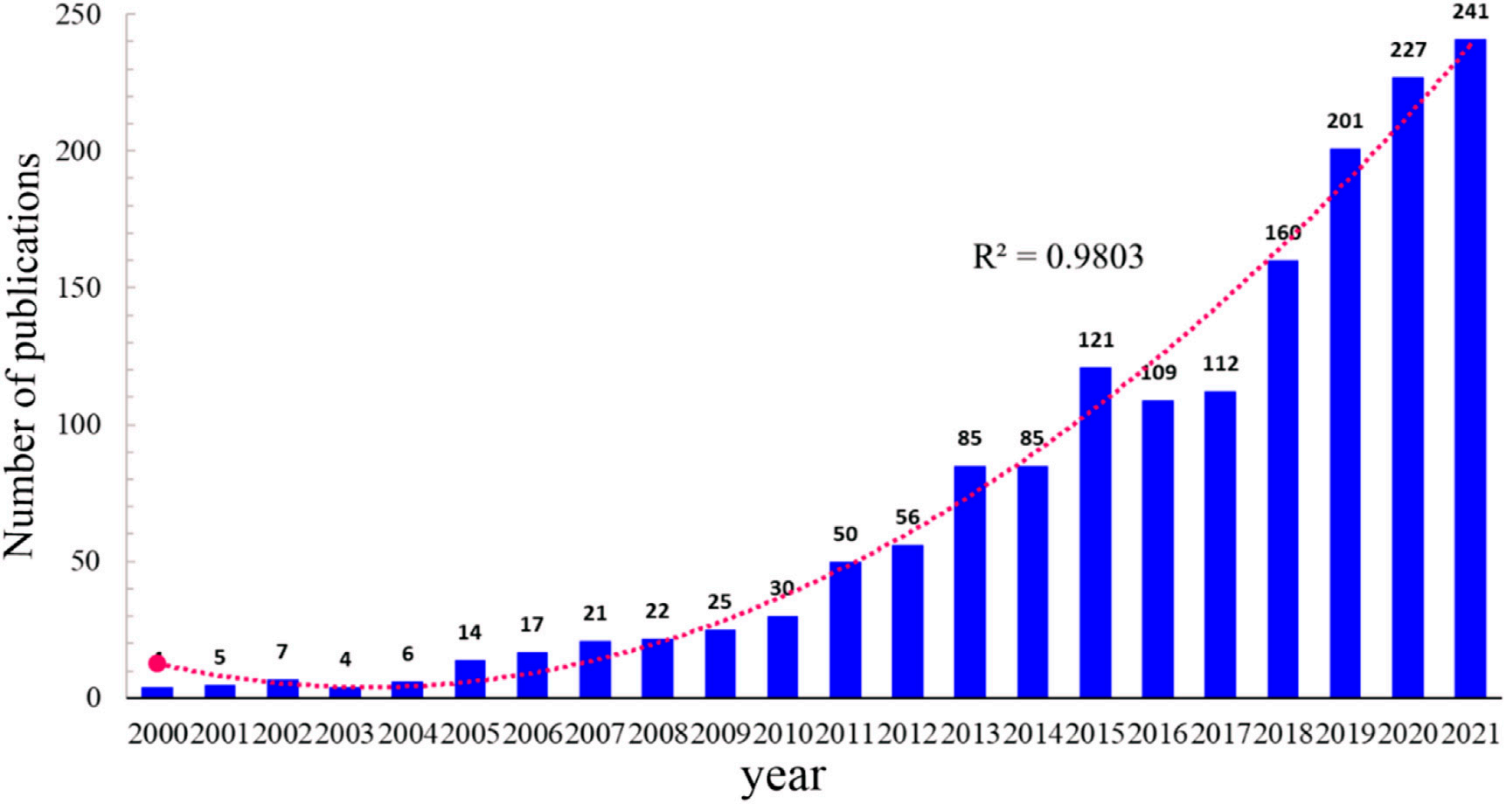
Number of publications by year and curve fitting of the annual growth trend of publications (*R* ^2^ = 0.9803).

The total NP over a given period can objectively and quantitatively reflect a field’s overall development trend. A total of 1,602 publications were chosen based on the defined search terms, including 1,289 original articles (80.46%) and 313 reviews (19.53%). The annual NP is shown in Figure 1. Despite some fluctuations, the NP increased from four in 2000 to 241 in 2021. Over the last 22 years, the growth rate remained relatively stable. Figure 1 also depicts the polynomial fitting curve for the publications’ total annual growth trend. The annual NP trended upward and was positively associated with the year of publication (*R* ^2^ = 0.9803). These findings indicate that the research and applications of herbal medicine in the treatment of CKD have attracted increasing attention.

### Country/region

**FIGURE 2:**
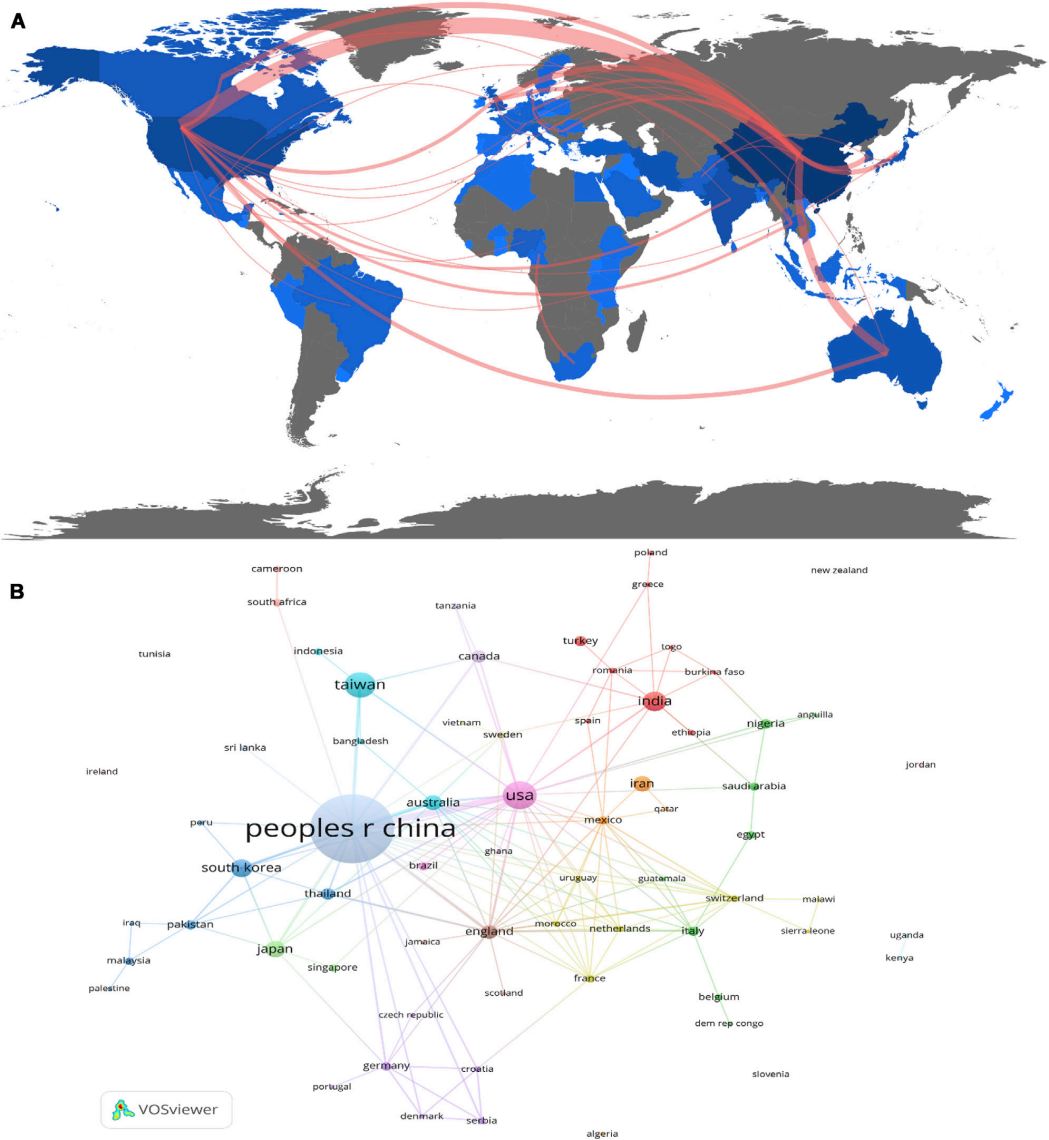
Regional distribution **(A)** and network map of countries/regions collaboration visualization map related to herbal medicine in the treatment of CKD **(B)**.

The publications were produced by 68 countries/regions, and the 10 most influential countries/regions are listed in Table 1, along with their NP, NC, H-index, and average citations per item (AC). China was the leading country in terms of NP (64.8%, 1,038/1,602), followed by the United States (10.0%,161/1,602) and India (6.6%,106/1,602). The top three countries also have the highest NC and H-index. As indicated in Figure 2A, the top 10 countries/regions were distributed across four continents, of which six were located in Asia. Figure 2B illustrates the countries/regions’ coauthorship network. China, the most productive country, showed the most cooperation with other countries/regions, followed by the United States, India, South Korea, and Iran. These countries/regions had close collaborations with other countries/regions, indication that theyhave more research exchanges and collaborations.

**TABLE 1:** Topmost 10 productive countries/regions in the field of herbal medicine to control CKD.

Rank	Country	NP^a^	NC^b^	AC^c^	H-index
1	China	1038	14,248	15.49	56
2	United States	161	6490	40.54	38
3	India	106	1981	18.91	23
4	South Korea	70	835	12.07	16
5	Iran	59	690	11.86	15
6	Japan	52	752	14.60	16
7	Australia	35	1039	30.00	16
8	Canada	34	643	18.97	14
9	England	27	1081	40.22	13
10	Pakistan	20	169	8.45	8

a NP, Total number of publications.

b NC, Total number of citations.

c AC, Average citations per item.

### Institutions

**FIGURE 3:**
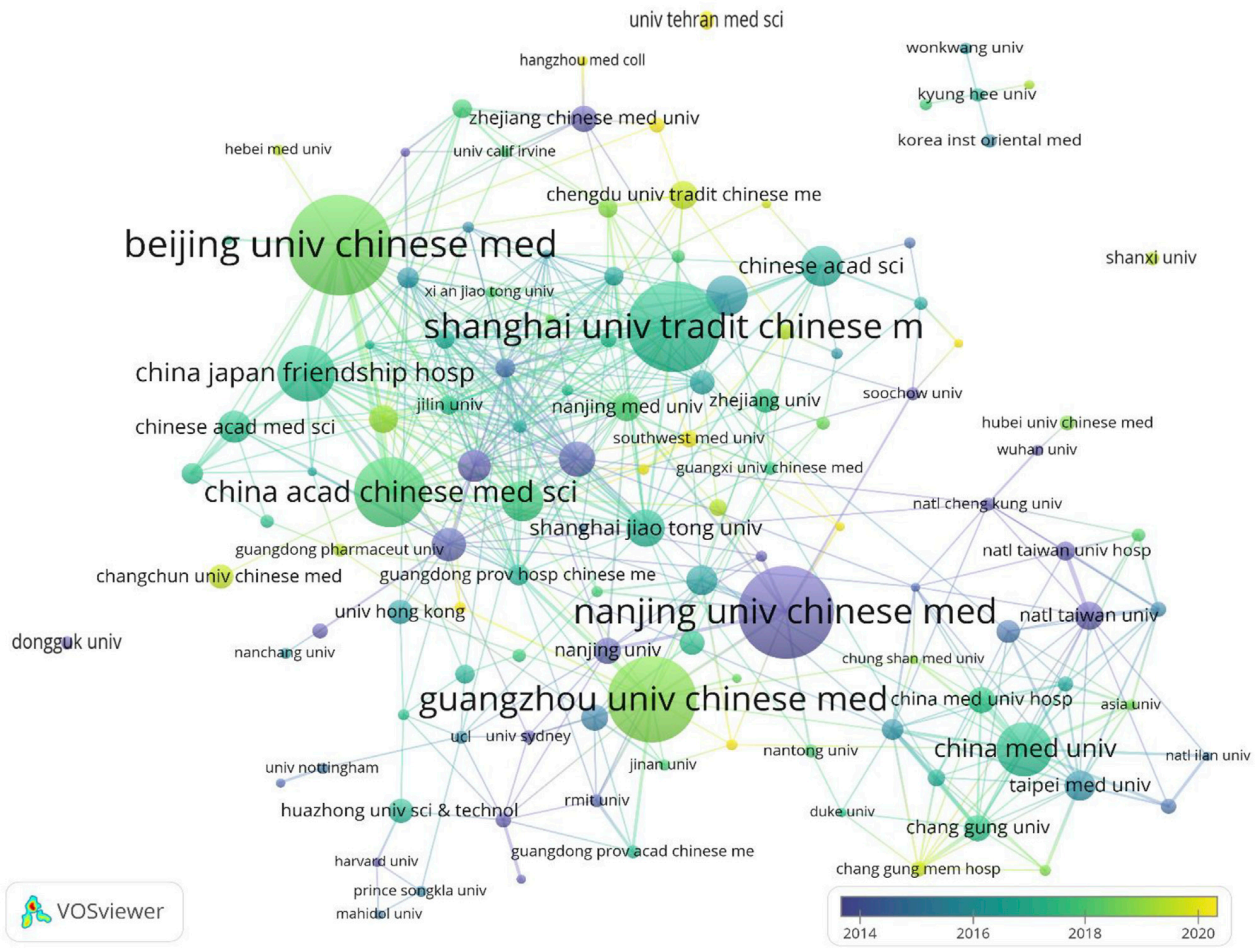
Institutions coauthorship network visualization map.

More than 1,330 institutions contributed to this field, among which 122 institutions produced more than four papers. Table 2 summarizes the 10 most influential institutions with the highest NP related to herbal medicine for the treatment of CKD. The Beijing University of Chinese Medicine had the highest NP (83), followed by Guangzhou University of Chinese Medicine (77) and Nanjing University of Chinese Medicine (72). The most significant AC scores were obtained from Fudan University (20.87), followed by Nanjing University of Chinese Medicine (17.03) and Shanghai University of Traditional Chinese Medicine (15.70). Figure 3 depicts the institutional coauthorship network, in which the frequent collaborating institutions include the Beijing University of Chinese Medicine, China-Japan Friendship Hospital, Shanghai University of Traditional Chinese Medicine, and China Academy of Chinese Medical Sciences. Interestingly, the most productive institutions are mainly specialized medical universities and have a high frequency of collaboration network with other institutions.

**TABLE 2:** Topmost 10 productive institutions in the field of herbal medicine to control CKD.

Rank	Affiliation	Country	NP^a^	NC^b^	AC^c^	H-index
1	Beijing University of Chinese Medicine	China	83	758	9.64	13
2	Guangzhou University of Chinese Medicine	China	77	463	6.91	13
3	Nanjing University of Chinese Medicine	China	72	1186	17.03	20
4	Shanghai University of Traditional Chinese Medicine	China	66	987	15.70	19
5	China Academy of Chinese Medical Sciences	China	60	797	13.53	15
6	Guang’ Anmen Hospital	China	39	558	14.62	11
7	China Japan Friendship Hospital	China	35	335	10.94	11
8	Capital Medical University	China	31	429	13.94	9
9	Shanghai Jiao Tong University	China	31	389	12.77	12
10	Fudan University	China	30	622	20.87	13

a NP, Total number of publications.

b NC, Total number of citations.

c AC, Average citations per item.

### Authors and cocited authors

**FIGURE 4:**
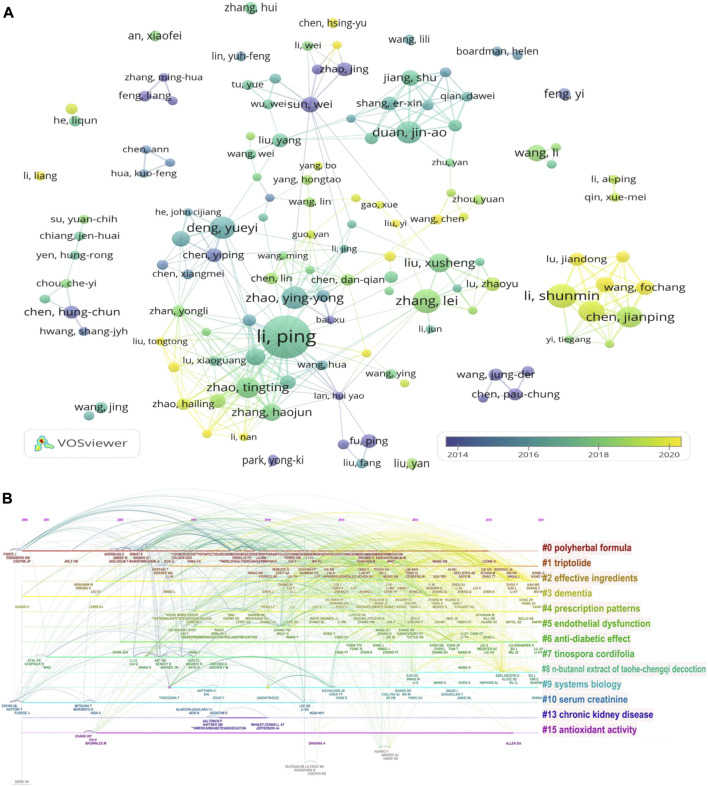
**(A)** Author coauthorship network visualization map. **(B)** Timeline view of cocited authors cluster analysis.

Over 5,920 authors have authored publications in this field. Among them, 129 authors contributed at least four papers to this collection. Table 3 summarizes the 10 most active and productive authors. Ping Li (China-Japan Friendship Hospital) was the most prolific author (NP = 28, NC = 319) followed by Xusheng Liu (Guangzhou University of Chinese Medicine) and Yueyi Deng (Shanghai University of Traditional Chinese Medicine). Yingyong Zhao (Northwest University Xi’an) had the highest AC and *H* index. A network of coauthorship was produced using VOSviewer (Figure 4A **)**. Ping Li and Tingting Zhao are the most cooperative authors. The data suggest that the researchers’ cooperation is relatively loose, and the team’s radiation scope is small.

**TABLE 3:** The top 10 active authors in the field of herbal medicine to control CKD.

Rank	Author	Affiliation	Country	NP^a^	NC^b^	AC^c^	H-index
1	Li Ping	China-Japan Friendship Hospital	China	28	319	12.96	11
2	Liu Xusheng	Guangzhou University of Chinese Medicine	China	24	171	8.08	7
3	Deng Yueyi	Shanghai University of Traditional Chinese Medicine	China	19	453	24.84	10
4	Zhao Tingting	China-Japan Friendship Hospital	China	17	194	12.71	9
5	Li Shunmin	Guangzhou Univ Chinese Med	China	15	78	7.13	8
6	Zhao Yingyong	Northwest University Xi’an	China	15	997	69.93	13
7	Duan Jin-ao	Nanjing University of Chinese Medicine	China	14	221	15.93	8
8	Chen Jianping	Guangzhou University of Chinese Medicine	China	12	75	7.92	8
9	Liu Xinhui	Guangzhou University of Chinese Medicine	China	12	74	8.17	8
10	Zhang Lei	Guangzhou University of Chinese Medicine	China	10	56	5.90	4

a NP, Total number of publications

b NC, Total number of citations.

c AC, Average citations per item.

Cocited authors are two or more authors who are simultaneously cited in one or more papers. These authors are related *via* cocitations. Among the 43,062 cocited authors, 154 had at least 50 citations. CiteSpace was used to cluster cocited authors to identify research hotspots, and the “log-likelihood ratio” algorithm was employed for extracting group inscriptions. As shown in Figure 4B, the node size and color on the horizontal line represent the NP and year range of occurrence, respectively. Purple outlines indicate articles with significant betweenness centrality, and red nodes indicate frequently cited references (Kaur et al., 2017). In addition, the uppermost line shows the timeline for different fields, and the number of longitudinal lines describe the distinct categories of herbal medicine to control CKD research, which are arranged vertically in descending order of the cluster’s size; the smaller the number of clusters is, the more authors are included, and each cluster is composed of multiple closely related words. Early research on herbal medicine for CKD control was conducted on polyherbal formula. Figure 4B shows the timeline view of cocited author clusters, and their cluster labels are located on the right. Based on the cluster summary, the polyherbal formula has been the focus of current research. The popular themes that have emerged in recent years also include Polyherba formula, triptolide, effective ingredients, dementia, prescription patterns, endothelial dysfunction, anti-diabetic effect, Tinospora cordiflolia, n-butanol extract of taohe-chenqi decoction, systems biology, serum creatinine, chronic kidney disease, and antioxidant activity.

### Journals and cocited journals

A total of 355 scholarly journals have published papers on herbal medicine in the treatment of CKD. A total of 138 journals had more than two publications. Table 4 lists the top 10 most productive journals, their publishers, ISSN, NP, the impact factor (IF), NC, and *H* index. Most of the journals specialize in pharmacotherapy. *Journal of Ethnopharmacology* was the most prolific journal (NP = 155, NC = 4,486, H-index = 36) followed by *Evidence Based Complementary and Alternative Medicine* (NP = 106, NC = 686) and *Frontiers in Pharmacology* (NP = 77, NC = 665)*. Biomedicine Pharmacotherapy* had the highest IF (7.419), followed by the *Phytomedicine* (6.656) and *American Journal of Chinese Medicine* (6.005).

**TABLE 4:** The top 10 most productive journals in the field of herbal medicine to control CKD.

Rank	Journal	ISSN	Country	NP^a^	IF-2021^b^	NC^c^	H-index
1	Journal of Ethnopharmacology	0,378–8741	Ireland	155	5.195	4484	36
2	Evidence Based Complementary and Alternative Medicine	1,741–427X	England	106	2.650	686	13
3	Frontiers in Pharmacology	1,663–9812	Switzerland	77	5.988	665	15
4	Medicine	0,025–7974	United States	44	1.817	123	6
5	BMC Complementary and Alternative Medicine	1,472–6882	England	35	4.782	524	14
6	Plos One	1,932–6203	United States	29	3.752	752	18
7	Chinese Journal of Integrative Medicine	1,672–0415	China	27	2.626	164	7
8	Phytomedicine	0,944–7113	Germany	26	6.656	535	15
9	Biomedicine Pharmacotherapy	0,753–3322	France	21	7.419	385	11
10	American Journal of Chinese Medicine	0,192–415X	United States	18	6.005	295	12

a NP, Total number of publications.

b IF, Impact factor.

c NC, Total number of citations.

**FIGURE 5:**
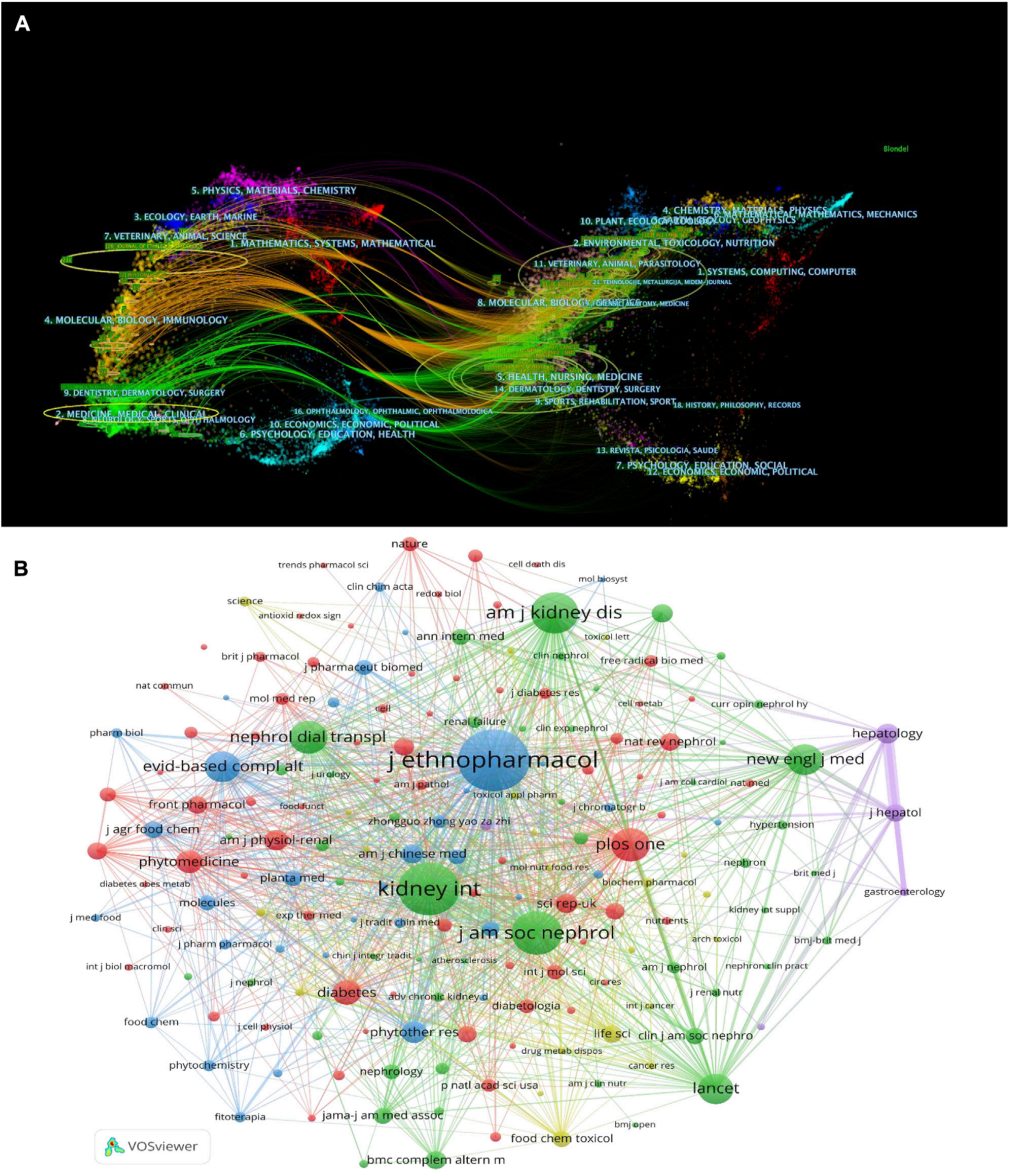
**(A)** The dual-map overlay of journals on herbal medicine for chronic kidney disease. **(B)** Citations journals co-occurrence analysis.

In co-cited journals, two or more journals are cited concurrently by researchers. Among the 9,186 cocited journals cited by the retrieved papers, 182 had at least 50 cocitations. The dual-map overlay of journals demonstrated relationship distribution among journals, with citing journals on the left and cited journals on the right, and the colored paths between them suggest the cited relationships. The green path in Figure 5A indicates that the documents published in health/nursing/medicine journals are often cited by medicine/medical/clinical journals. Figure 5B visualizes the journals of clusters in cocited journals network map. Each journal in the visualization has different size that indicates its frequency. *Journal of Ethnopharmacology*, *Kidney International*, *Journal of the American Society of Nephrology*, *American Journal of Kidney Diseases,* and *Nephrology Dialysis Transplantation* were the five most frequently and centrally cited journals.

### References

**FIGURE 6:**
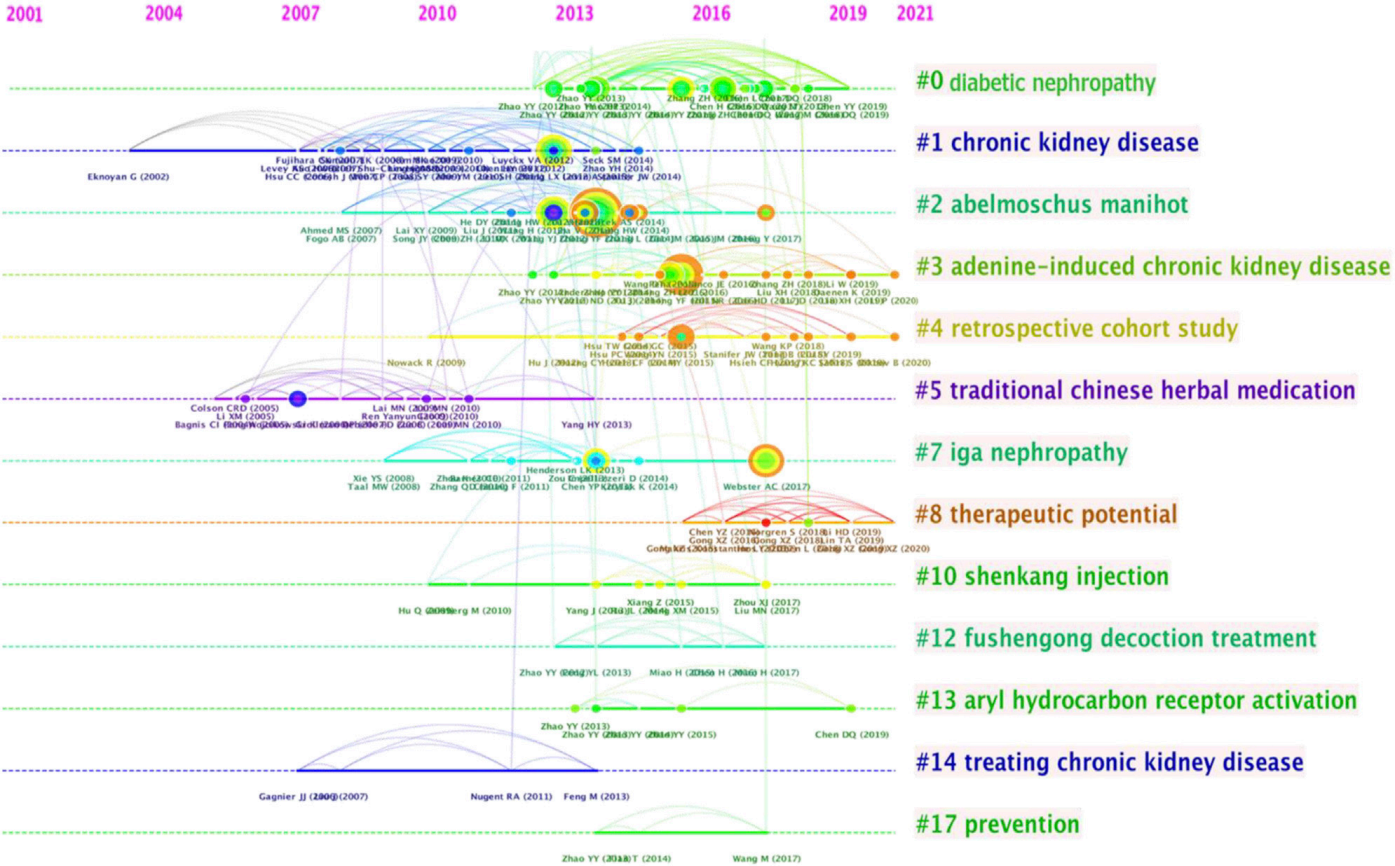
Timeline view of cocited references cluster analysis.

Among the 45,867 cocited references cited by the retrieved papers, 210 had at least nine cocitations. Figure 6 shows the timeline of the cocited reference clusters that was used to divide the papers into different clusters. Cluster 0 included the most references, and it mainly focused on diabetic nephropathy, indicating that this topic was closely related to herbal medicine to control CKD research and needs further mining. The popular themes that emerged in *Abelmoschus Manihot*, adenine-induced chronic kidney disease, retrospective cohort study, traditional Chinese herbal medication, IgA nephropathy, therapeutic potential, Shenkang injection, Fushengong decoction treatment, aryl hydrocarbon receptor activation, treating CKD, and prevention were also included this field.

### Research hotspots

**FIGURE 7:**
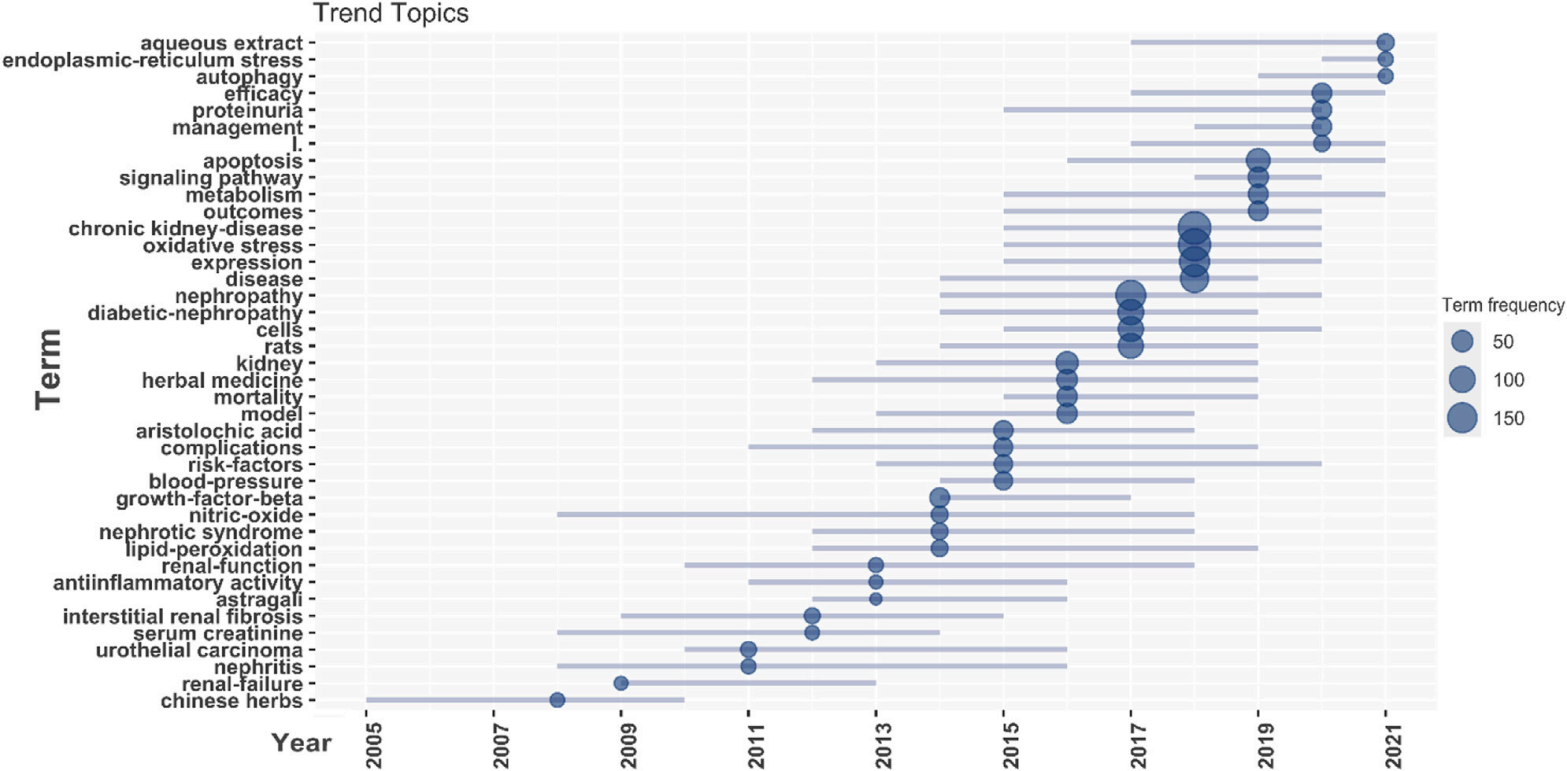
Frequency of keywords occurrence over time in the topic of herbal medicine to control CKD.

The keywords filter the research content of the literature and represent the theme of the article. When a keyword appears frequently, it reveals the distribution of scientific research topics and the hotspots and trends of research. Keyword analysis can also determine the time when a keyword with a change in frequency first appeared in a node, thereby defining the research field’s boundaries. Between 2000 and 2021, 8,078 keywords were used, in which 131 keywords occurred for more than 20 times. The Bibliometrix and Bibliophagy installation packages in the R tool were adopted to count the author keywords and draw a trend topic of the top 40 keywords in this area. Figure 7 shows that oxidative stress, chronic kidney disease, expression, and diabetic nephropathy account for the highest frequency of the total keywords, while, aqueous extract, endoplasmic-reticulum stress, and autophagy are new high-frequency keywords in the past 5 years.

**FIGURE 8:**
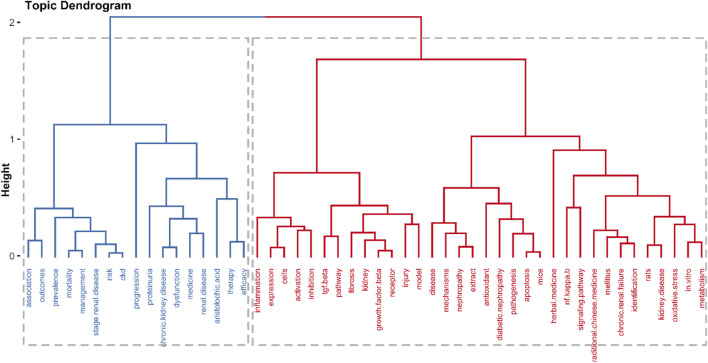
Tree dendrogram of hierarchical cluster analysis of keywords in the field of herbal medicine treatment for CKD.

Clustering analysis in literature metrology, which is based on the frequency of two or two keywords appearing, uses statistical methods to simplify the complex keyword mesh relationship into a few relatively small groups of classes. As shown in Figure 8, hierarchical clustering was used to cluster the keywords; the two others with the uppermost similarity scale were clustered to generate another cluster. Then, a tree dendrogram that details the correlation and decorrelation between the keywords was generated. The plot revealed two clusters represented as cluster 1 (blue) and cluster 2 (red). Cluster 1: mainlyshows the general epidemiological survey and pathological findings of CKD. Cluster two indicates the mechanism of action and key regulators of herbal medicine in the treatment of CKD.

## Discussion

Herbal medicine has unique advantages in the treatment of chronic diseases, and the potential value associated with herbal medicine application has attracted attention (Zhong et al., 2013). The development of herbal medicine as a supplement in combination with western medicine for the treatment of CKD has become an emerging research area (Lu et al., 2020). Improving knowledge of herbal medicine application in CKD is crucial for drug discovery and clinical application research. In the present, the application of herbal medicine in the treatment of CKD research status from 2000 to 2021 was analyzed for the first time based on the quantity of publications, cocitation analysis, and keyword analysis.

In the past 22 years, trends in the NP about “herbal medicine to control CKD” have substantially, increased. Therefore, the broad pharmacological activities of herbal medicine are attractive to researchers and will be a significant research field (Figure 1). According to the polynomial fitting curve, the annual NP trended generally upward and increased in the latter half of the period, particularly after 2012. The rapid economic growth and investment in scientific research indirectly led to an annual increase in the NP, which may be the main reason for this result. From 2000 to 2021, the most productive country in terms of publishing studies on herbal medicine for CKD control was China (Figure 2). In China, the application of herbal medicine in the treatment of nephropathy has a history of thousands of years, but the research on its pharmacological mechanism and clinical application was performed slightly later but developed quickly, making it the first in the NP. However, compared with other medical disciplines, the NC and *H* index are low. Therefore, ethnopharmacology scholars and institutions need further studies to improve their research output.

Analysis of collaboration networks revealed collaborations between different countries. China has the most cooperation with United States, England, Denmark, Canada, Thailand, South Korea, Australia, South Africa, Japan and other countries. Among them, China and the United States have the thickest connection, indicating that the cooperation between the two countries is the strongest. However, China has less cooperation with India, Egypt, New Zealand, Turkey, Iran and other countries. The possible reason is that these countries have less research in this field and their research foundation is weak. Therefore, in future research, China should strengthen cooperation with more countries to further promote the development of herbal medicine in the treatment of CKD.

Eight of the top 10 productive institutions from Chinese Medicine University, and five of the top 10 productive authors were from Guangzhou University of Chinese Medicine, suggesting that the Chinese Medicine University was the pioneer in the research on herbal medicine for CKD control (Table 3). Ping Li was the most prolific researcher in this field and focused mainly on the mechanism, efficacy, and safety evaluation of herbal medicine in CKD treatment (Li et al., 2012; Li et al., 2021). Yingyong Zhao was the most cited author publishing articles on the herbal medicine treatment for CKD and focused mainly on metabolomics and traditional Chinese medicine against renal interstitial fibrosis research. In addition, other frequently cited works illustrated multiple mechanisms of herbal medicine in the treatment of CKD (Figure 4). Among the top 10 journals that published studies on the herbal medicine for CKD control, five journals had an IF greater than 5. The number of articles published in these journals accounted for 7.7% of the total publications included in this study. Therefore, publishing research on herbal medicine for CKD control in high-quality journals is a challenge. To date, the most relevant subjects of herbal medicine for CKD control include ethnopharmacology, pharmacology, integrative medicine, and molecular biology, which may continue to be the main subjects of focus in future studies (Figure 5). Articles with a high NC were published in journals with a high-IF, indicating that these journals have published a greater number of potential breakthroughs in this field. As a result, researchers interested in this field focus on recent publications in these journals.

Hot spot analysis helps in exploring research frontiers and trends. Based on the analysis of the keyword map (Figure 7), the current research hotspot in the field is gradually shifting from the research of clinical diagnosis, and pathological to signaling pathways and regulatory indicators. The various active functions of herbal medicine in the prevention and treatment of CKD do not exist in isolation, and they exist synergistic or causal relationships between them. Herbal medicines and polyherbal formulas may protect the kidneys in a different way*.* Oxidative stress is a central link in the pathogenesis of CKD, and various traditional Chinese medicines and natural products (e.g., Abelmosk capsule and Shenkang Injection et al.) can significantly regulation NrF2/ARE and NF-κB pathway signaling pathways to improve the oxidative stress state of CKD (Cai et al., 2018; Ji et al., 2018; Feng et al., 2019; Fu and Hu, 2019; Yu et al., 2022)*.* Micro-inflammatory state is an important factor in promoting kidney disease progression. Herbal medicines such as *Rheum officinale* and curcumin could intervene in the damaged cells to interfere with the micro-inflammatory state and protect renal function (Zou et al., 2020). Renal fibrosis is one of the most important pathological manifestations of chronic nephritis as the disease progresses to the end stage, and it causes chronic nephritis (Wu et al., 2015; Luo et al., 2021a). The decisive factor of renal failure, traditional Chinese medicine (curcumin, rhubarb, etc.,) and herbal preparations (Shenqi pills, Huangqi-Danshen decoction, etc.,) can effectively slow down the process of renal fibrosis by regulating TGF-β/Smad, downstream signaling pathways, and Notch 1/jagged 1 (Chen et al., 2017; Liu et al., 2019). Furthermore, podocyte injury involves all stages of CKD, thus reducing podocyte damage and apoptosis is an important link in delaying the development of CKD (Cao et al., 2019; Wang et al., 2018). No effective treatment method is currently available for podocyte injury in western medicine, while some traditional Chinese medicines and compound prescriptions have anti-podocyte injury effects (Wang et al., 2018). Overall, these recent research hotspots may make herbal medicine an exciting clinical candidate for the treatment or prevention of CKD.

Currently, the most successful and extensive research on the use of herbal medicine for CKD control is related to diabetic nephropathy. Of course, this is also an area of particular concern and efforts of various clinicians and scholars (Figure 6). The World Health Organization has listed more than 400 plants which are available used for herbal medicine treatment of diabetic nephropathy. Among them, *Astragalus memeranaceus*, *Salvia miltiorrhiza Lobed Kudzuvine* Root, *Panax notoginseng, Schisandra chinensis*and and other herbal formulas developed for the clinical treatment of diabetic nephropathy have achieved good clinical results (Wen et al., 2020). One of the main advantages of herbal medicine is the low side effects of these drugs, which has attracted various researchers to develop new molecules for the treatment of diabetes (Kumar et al., 2021). Of course, similar to other drug discovery, clinical application still faces many challenges. Bioavailability, active substance base or functional differences, safety, and clinical trials also need to be overcome. Undoubtedly, this will require researchers to demonstrate with their studies in the future.

### Limitations

This study is based on bibliometric analysis and visualization network analyses of the literature, which may improve the professional knowledge of the field’s development, status and trends, as well as understanding of academic frontiers among researcher. Additionally, this study employs the NC as an indicator, which may help scholars comprehend significant nodes in the trend in this field. Nonetheless, this study has some limitations. Above all, only articles published on English language and reviews from SCI-Expanded-indexed journals were included. Furthermore, some unavoidable deviations may be occurred because of the inability of R-bibliometrix, VOSviewer, and CiteSpace software to analyze the full text of publications. Additionally, some newly published excellent papers may be ignored due to lag and exhibits lower NC. We hope that future studies will look at more databases and obtain a complete picture of the research and application of herbal medicine in the treatment of CKD worldwide.

## Conclusion

The research on the complex mechanism of herbal medicine for CKD, the crosstalk between different types of CKD, and herbal medicine for CKD have shown important research value and broad application prospects, the research on the treatment of CKD with herbal medicine has been increasing yearly. By using CiteSpace, VOSviewer, and BiblioShiny for visual analysis, illustrated that China is the leading country in this research. Among the research institutions, the Beijing University of Chinese Medicine is the institution with the highest influence on achievements. Different countries and institutions need to strengthen cooperation and exchanges. Ping Li is an outstanding contributor to the field of herbal medicine treatment for CKD. Most of the articles concerning herbal medicine for CKD are cited from internationally influential journals, indicating that herbal medicine for CKD has received much attention. Current research on herbal medicine for CKD mainly focuses on diabetic nephropathy-related research, Balkan endemic nephropathy, and pharmacokinetic study, which will also be the focus of future research.

## Data Availability

The original contributions presented in the study are included in the article/Supplementary Material, further inquiries can be directed to the corresponding author.
